# An uncommon case of pheochromocytoma with positive biochemical workup and absence of tumor

**DOI:** 10.1530/EDM-25-0027

**Published:** 2025-09-02

**Authors:** Nicole Chan, Ronald P Kaufman, Nada Farhat, Grace Y Kim

**Affiliations:** ^1^ Division of Endocrinology and Metabolism, Albany Medical College, Albany, New York, USA; ^2^ Department of Urology, Albany Medical College, Albany, New York, USA; ^3^ Department of Pathology, Albany Medical College, Albany, New York, USA

**Keywords:** pheochromocytomas, rare diseases/syndromes, adrenal, neuroendocrinology

## Abstract

**Summary:**

Pheochromocytomas are rare neuroendocrine tumors derived from adrenal chromaffin cells that result in hyperactivity of the sympathetic nervous system. We present the case of a patient with biochemical evidence of pheochromocytoma, but surgical pathology revealed absence of tumor. This is an 80-year-old female with a past medical history of metastatic follicular lymphoma and hypertension with an incidental 1.4 cm right-sided adrenal nodule noted on a PET-CT scan. Her hypertension was treated with three different antihypertensive agents. Subsequent imaging with non-contrast CT of abdomen and pelvis showed a right adrenal incidentaloma with 16 Hounsfield units. Abdominal MRI with and without contrast revealed atypical signal loss on the out-of-phase imaging. Plasma normetanephrines were approximately 2.4 times higher than the upper limit of normal. Her urinary normetanephrines were higher than the upper limit of normal, suggestive of pheochromocytoma. The patient proceeded with robotically assisted laparoscopic right adrenalectomy and postoperatively required vasopressors. Surgical pathology showed adrenal cortical hyperplasia and medullary infarction associated with fibrosis. However, the noted phases of necrosis with predominant fibrosis match the time interval between clinical diagnosis and surgical management. Postoperative metanephrines normalized 4 weeks after surgery, indicating successful surgical resection of autonomous secretion of metanephrines. This is the only known case of biochemical evidence of pheochromocytoma with no histologic evidence of tumor.

**Learning points:**

- Biochemical evidence, clinical presentation and imaging studies of pheochromocytoma are crucial for its diagnosis.
- Due to the vascular nature of pheochromocytoma, there is a potential for the tumor to infarct.
- Plasma normetanephrines of greater than twice the upper limit of normal have high specificity for pheochromocytoma.

## Background

Pheochromocytomas are rare neuroendocrine tumors derived from adrenal chromaffin cells that result in hyperactivity of the sympathetic nervous system. In the United States, they occur in about 0.05–0.1% patients with sustained hypertension (1, 2). We present a case of a patient with biochemical evidence of pheochromocytoma, but surgical pathology revealed absence of tumor and residual normal medulla. To date, this is the only known case of biochemical presence of pheochromocytoma with normalization of metanephrines in the postoperative setting and complete absence of tumor in the surgical pathology evaluation.

The complete infarction and disappearance of the pheochromocytoma before surgery is extraordinarily rare. This case challenges the conventional expectation that these tumors will always be present at the time of surgical excision and how infarction can alter the typical pathological findings of pheochromocytoma. The observed necrosis and fibrosis evident in the surgical sample correlate with the timeline of diagnosis and surgery, which provides insights into the sequence of pheochromocytoma tumor infarction. This case also highlights the need for clinicians to remain vigilant when evaluating suspected pheochromocytoma cases that may evolve during its investigation and reinforces the role of biochemical monitoring in assessing treatment success. Given the rarity of spontaneous infarction and complete tumor disappearance, this case adds significant data to the growing body of knowledge on pheochromocytoma.

## Case presentation

This is an 80-year-old female with a history of metastatic follicular lymphoma and hypertension who presented for an evaluation of an incidental right-sided adrenal nodule noted on a routine PET-CT scan in March 2023 to evaluate her malignancy. The resulting PET-CT scan showed FDG maximum uptake measuring 5.1, suspicious for malignancy. Her hypertension was treated with lisinopril 40 mg, nifedipine 60 mg and atenolol 100 mg daily. She denied any history of weakness, pallor, headaches, palpitations or easy bruising.

## Investigation

**Figure 1:**
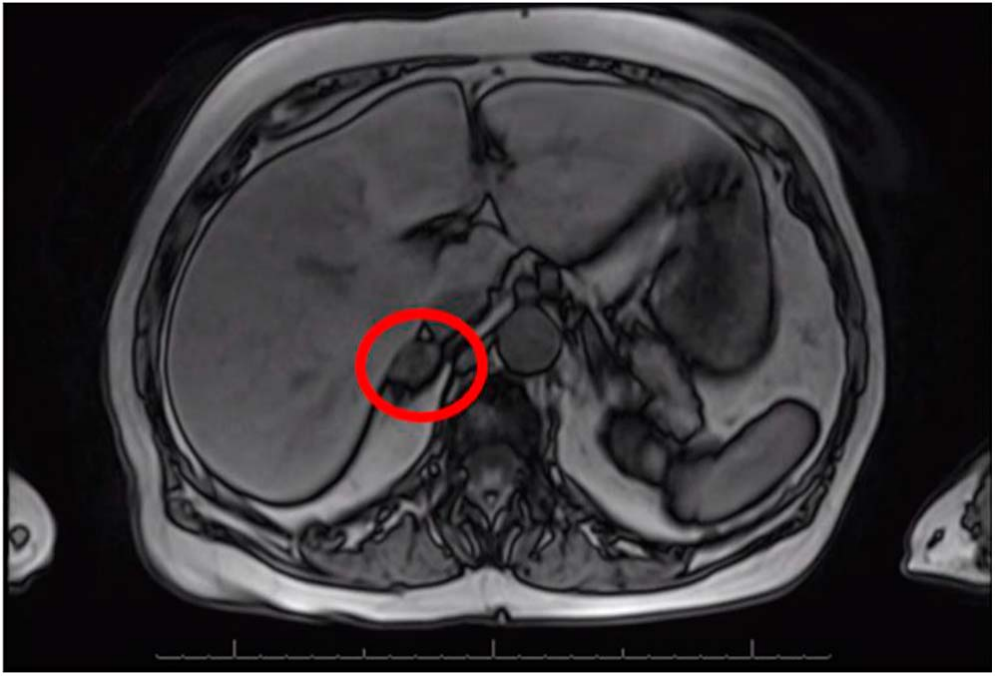
MRI abdomen. Axial T1, post-contrast and fat saturation.

**Figure 2:**
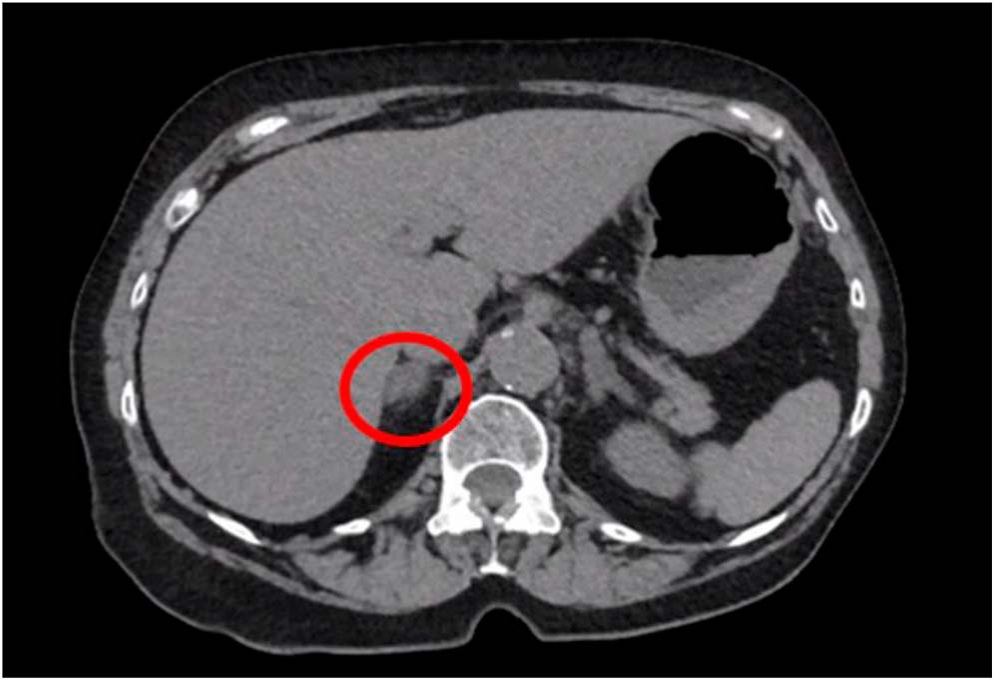
CT abdomen, pre-contrast phase.

Subsequently, abdominal MRI performed in April 2023 revealed atypical signal loss on the out-of-phase imaging (Fig. 1). The patient was referred to the endocrinology office in July 2023 to assess the functionality of the adrenal nodule. Biochemical testing for primary aldosteronism and Cushing’s syndrome was negative. Plasma normetanephrines were elevated at 675.7 pg/mL (2.24 nmol/L) (reference range: 0–285.2 pg/mL or < 0.9 nmol/L). The level of 24-h urinary normetanephrine was also elevated at 562 μg/day (1,865 nmoL) (reference range: 0–400 μg/day or 0–1,327 nmoL). In August 2023, CT abdomen and pelvis with and without contrast revealed a 1.4 cm right adrenal nodule of approximately 16 Hounsfield units (Fig. 2). These findings were suggestive of pheochromocytoma.

## Treatment

She was pre-treated with doxazosin 1 mg daily and afterward had a robotically assisted laparoscopic right adrenalectomy in September 2023. Postoperatively, the patient was hypotensive and required vasopressors briefly. She was eventually weaned off vasopressors and discharged on lisinopril 20 mg daily. Soon after discharge, the patient was complaining of hypotension. Her lisinopril was further decreased to 10 mg daily. She was able to maintain normal blood pressure on this dose.

## Outcome and follow-up

**Figure 3:**
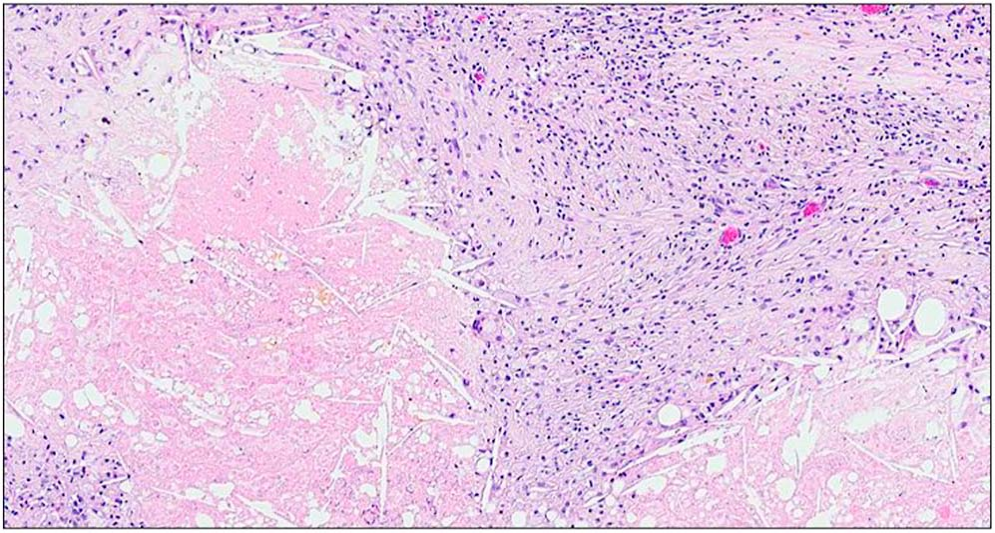
Surgical specimen of right adrenal mass. High power field. Adrenalectomy material showing predominantly infarcted adrenocortical tissue with hyperplastic changes.

**Figure 4:**
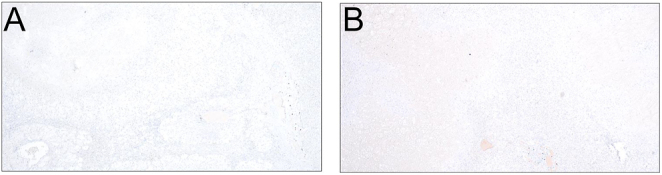
(A) Surgical specimen of right adrenal mass with negative chromogranin stain. (B) Negative synaptophysin stain.

Four weeks postoperatively, laboratory evaluation showed normalization of plasma normetanephrines to 268.2 pg/mL (0.83 nmol/L) (reference range: 0–285.2 pg/mL or < 0.9 nmol/L). Surgical pathology revealed benign adrenal cortical hyperplasia, medulla with fibrosis, infarction and hemosiderin deposition (Fig. 3). The specimen had negative staining for synaptophysin and chromogranin (Fig. 4A and B). There was no identifiable medullary neoplasm.

## Discussion

Pheochromocytomas are uncommon tumors originating from adrenal chromaffin cells that result in hyperactivity of the sympathetic nervous system (1, 2). The classic triad that is typically associated with pheochromocytoma, such as headaches, diaphoresis and palpitations, had been cited to occur in up to 28% (3). Therefore, the triad is less common than is often perceived. Pheochromocytomas are highly vascular tumors as angiogenesis is a critical step in tumor growth (4). Given this characteristic, it has the potential for infarction in certain circumstances.

This case shows classic radiologic and biochemical evidence of pheochromocytoma but unusual situation of recent adrenal infarction adjacent to hyperplastic adrenal cortex with obliteration of the tumor. At the start of the patient’s evaluation, MRI was performed due to concern for malignancy. The gradient-echo sequences and the appearance after contrast administration did not show typical signal loss that would make a diagnosis of a benign adenoma. The following abdominal CT scan revealed more information through the pre-contrast density measurements of 16 Hounsfield units. Current recommendations suggest testing for biochemical evidence for pheochromocytoma with findings of pre-contrast density greater than 10 Hounsfield units, as the sensitivity for excluding pheochromocytoma when the density is less than 10 Hounsfield units has been cited to be 99.6% (5). Consequently, the patient’s plasma normetanephrines were greater than twice the upper limit of normal, which does not occur in patients without pheochromocytoma with 100% specificity (6). The patient’s elevated 24-h normetanephrines further strengthen the suspicion for pheochromocytoma. The patient was screened for substances that may produce false positives of her elevated serum and urine normetanephrines, such as tricyclic antidepressants, selective serotonin reuptake inhibitors, sympathomimetic drugs or excess alcohol or caffeine use, that may contribute to abnormal biochemical findings (7, 8). None of these were found to be the case to contribute to her abnormal biochemical findings. Prior to the robotically assisted laparoscopic right adrenalectomy, the patient’s systolic blood pressure ranged from 140 to 155 mmHg and her diastolic blood pressure ranged from 80 to 82 mmHg. Upon initiation of doxazosin 1 mg daily, she achieved normotensive blood pressure. The relatively low dose of doxazosin was likely sufficient due to the concurrent use of her baseline antihypertensive regimen and the presumed tumor infarction, both of which may have contributed to a reduced catecholamine burden immediately leading up to her surgical intervention. Notably, the patient had developed profound hypotension, a well-recognized hemodynamic response following removal of pheochromocytoma. Interestingly, the pathological examination revealed complete obliteration of the medullary tissue. The total disappearance of the tumor is extraordinarily rare. Moreover, the normalization of plasma normetanephrines indicates the successful surgical removal of the source of excess metanephrines. The preoperative hormonal levels, combined with the complete absence of neoplastic tissue, suggest that tumor infarction may have occurred between the biochemical confirmation of pheochromocytoma, subsequent imaging and eventual surgery. The observed phases of necrosis, with predominant fibrosis, align with the time interval between clinical diagnosis and surgical management. This case highlights the critical role of biochemical evaluation in both the diagnosis and treatment of pheochromocytoma. It also emphasizes the variability in the pathological course of pheochromocytomas, underlining that not all cases follow a predictable pattern. By presenting this case, we hope to contribute to the growing body of knowledge on pheochromocytoma and its potential for atypical presentations.

## Declaration of interest

The authors declare that there is no conflict of interest that could be perceived as prejudicing the impartiality of the work reported.

## Funding

The authors did not receive any specific grant from any funding agency in the public, commercial or not-for-profit sector.

## Patient consent

Written informed consent for publication of their clinical details and clinical images was obtained from the patient.

## Author contribution statement

All authors made individual contributions to authorship. NC, RK and GK were involved in the diagnosis and management of this patient and manuscript submission. NF was involved in histopathological diagnosis and preparation of histology images. RK was responsible for the patient’s surgeries. All authors reviewed and approved the final draft.
